# Supplementary figures and images for: Adsorption of thallium(I) on rutile nano-titanium dioxide and environmental implications
Source: PeerJ. 2019 May 16;7:e6820. doi: 10.7717/peerj.6820 (PMC6526007; doi:10.7717/peerj.6820)

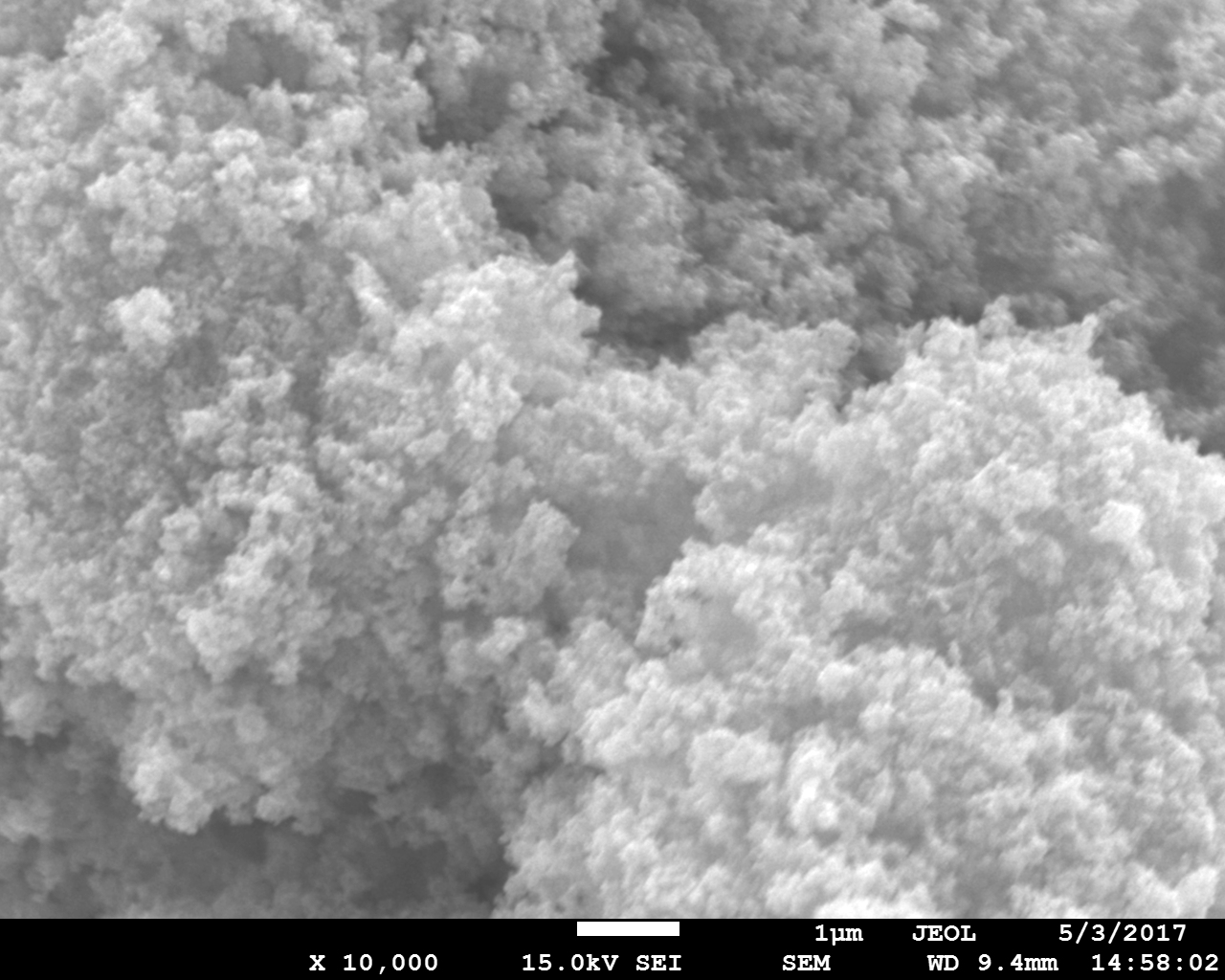

Supplement: Supplemental Information 1 [file peerj-07-6820-s001.zip › Supplemental Files/sem/20170503-18-26/17-2σÉ╕ΘÖäσëì.jpg]

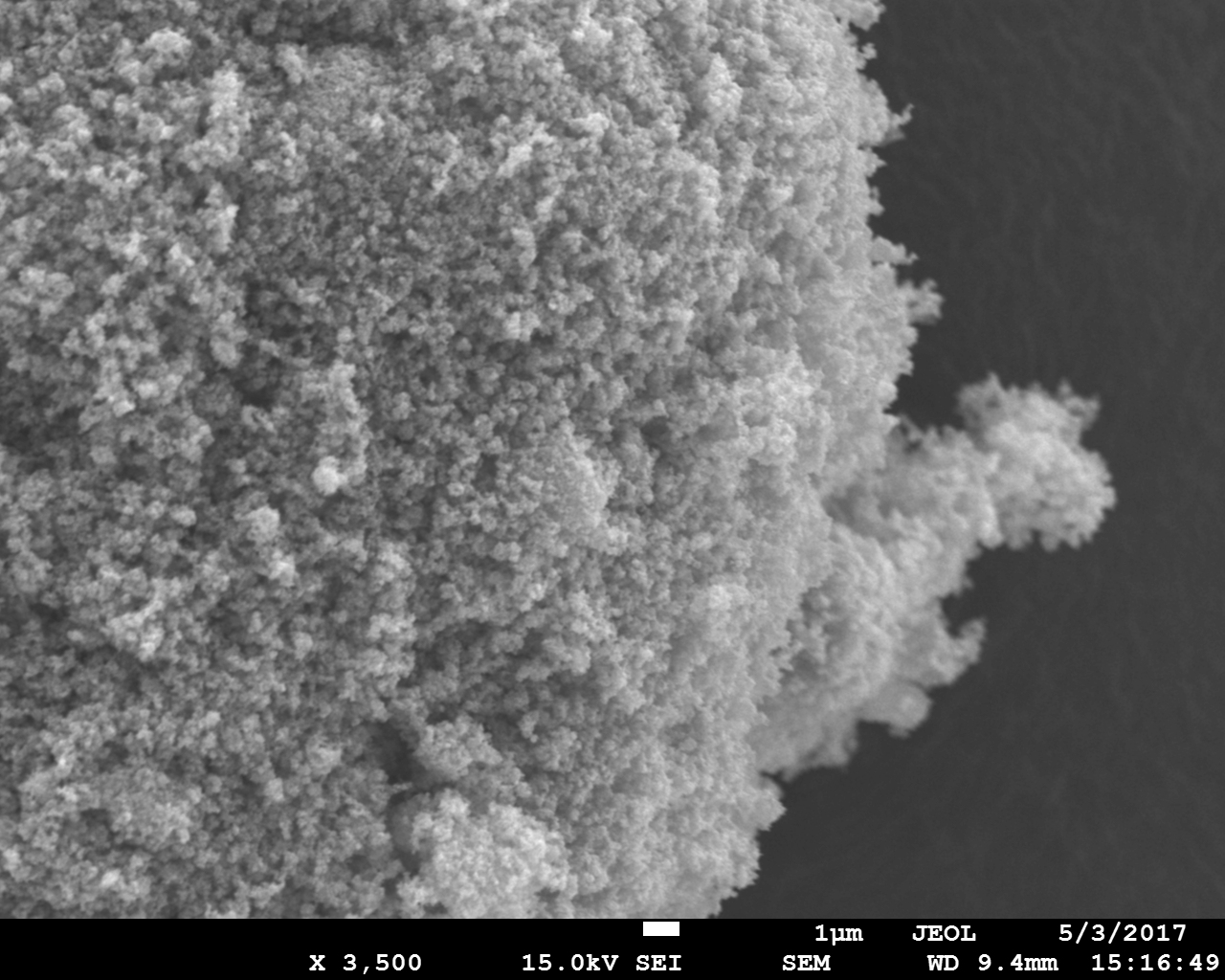

Supplement: Supplemental Information 1 [file peerj-07-6820-s001.zip › Supplemental Files/sem/20170503-18-26/17-4σÉ╕ΘÖäσëì.jpg]

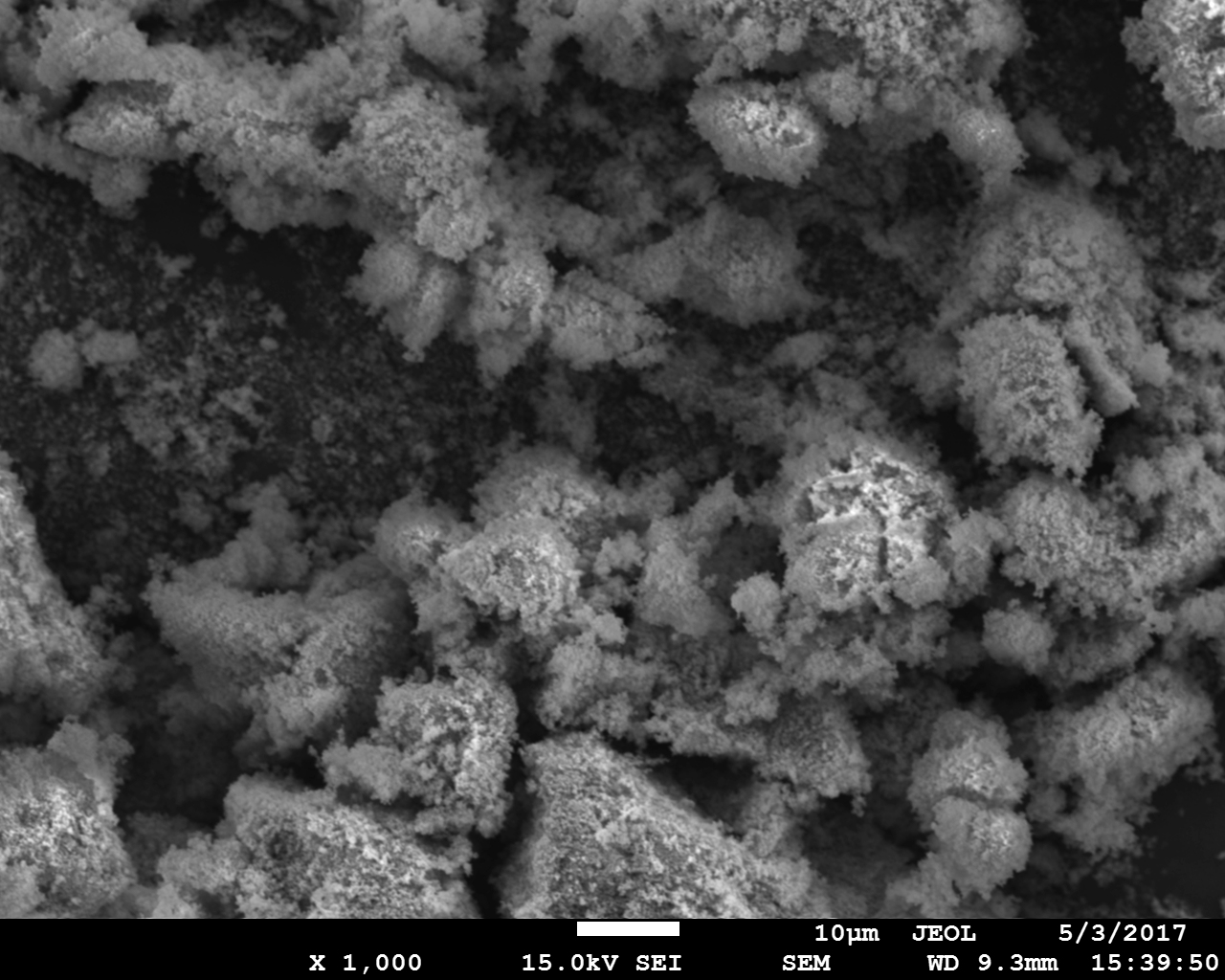

Supplement: Supplemental Information 1 [file peerj-07-6820-s001.zip › Supplemental Files/sem/20170503-18-26/18-4.jpg]

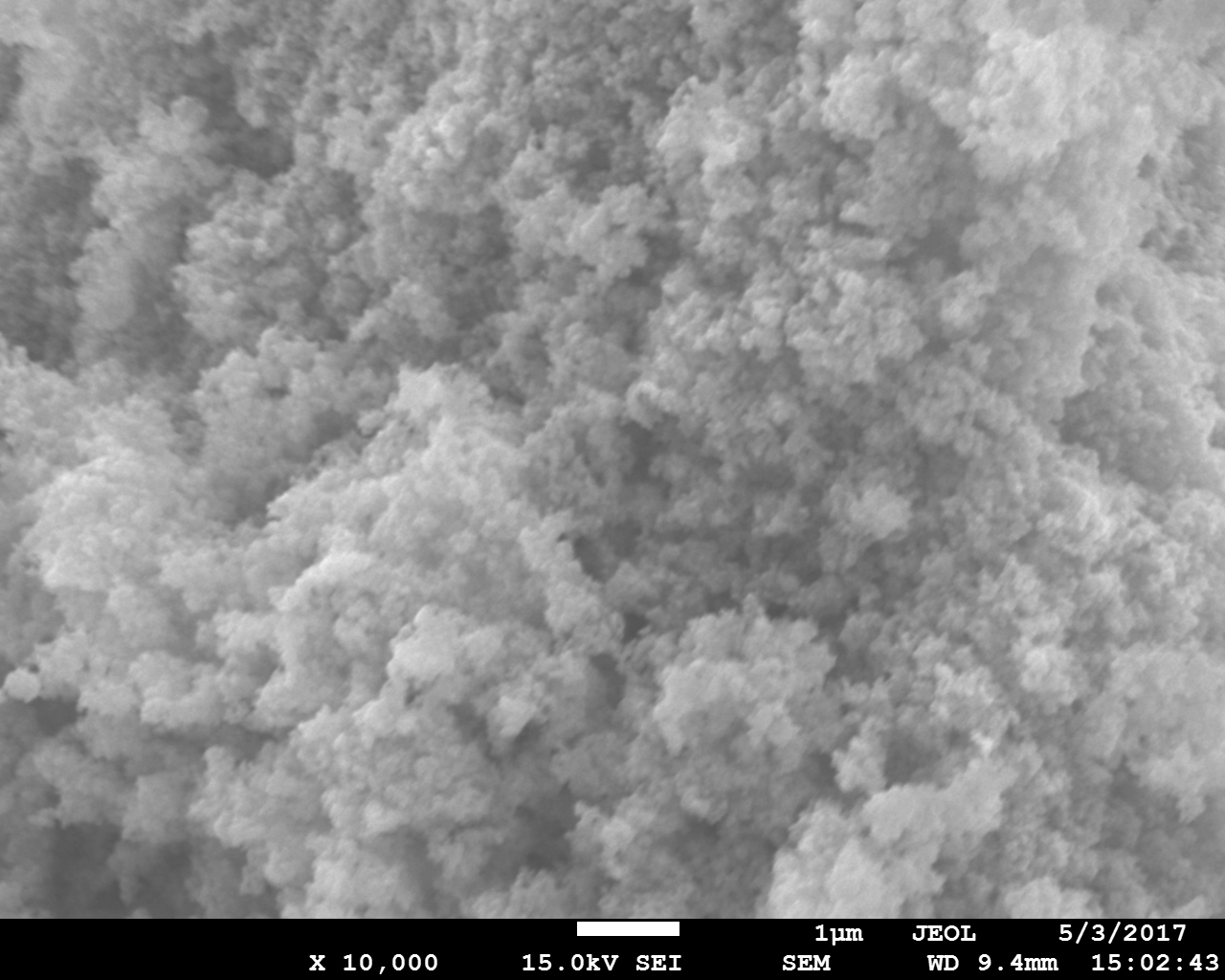

Supplement: Supplemental Information 1 [file peerj-07-6820-s001.zip › Supplemental Files/sem/20170503-18-26/17-3σÉ╕ΘÖäσëì.jpg]

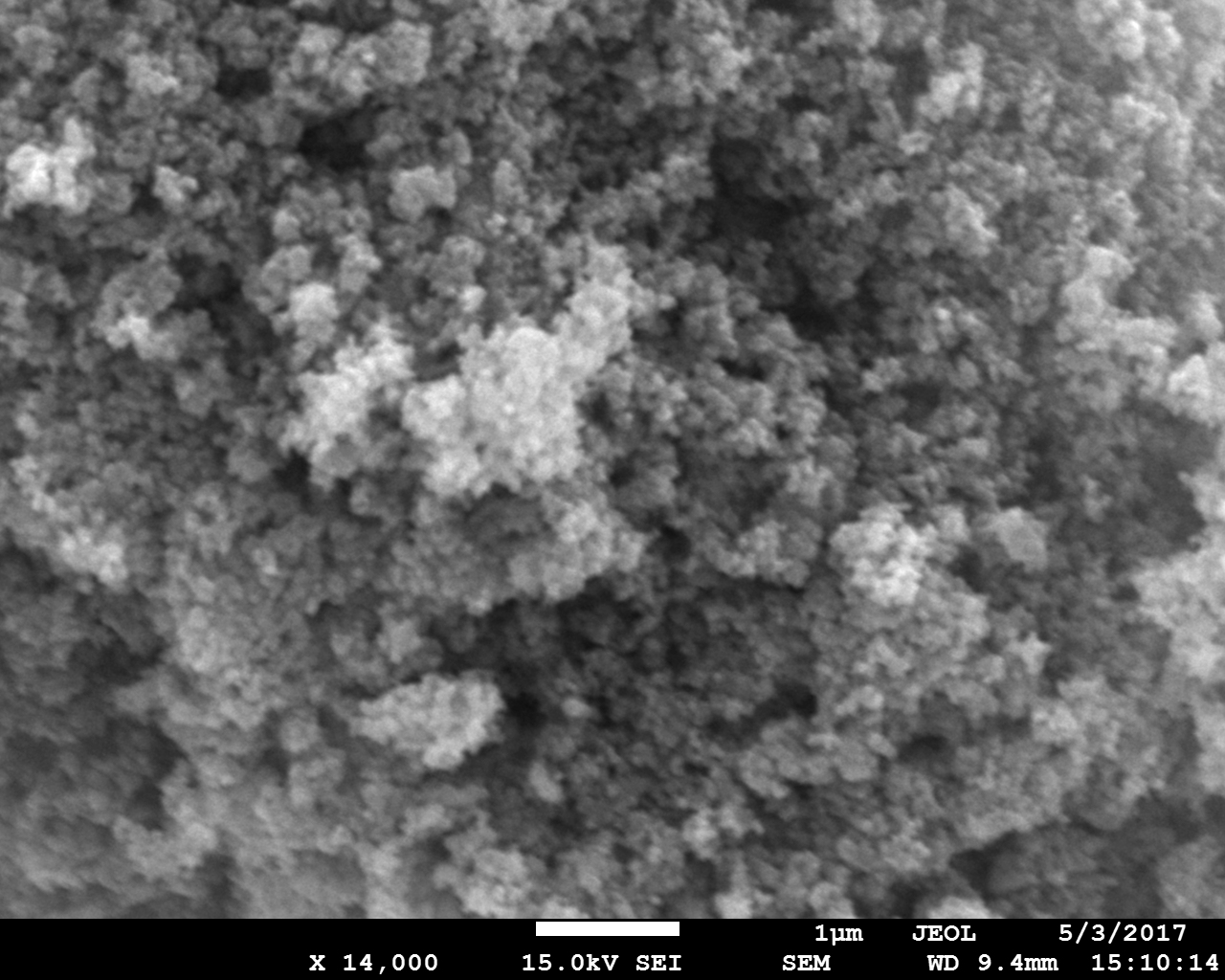

Supplement: Supplemental Information 1 [file peerj-07-6820-s001.zip › Supplemental Files/sem/20170503-18-26/18-2.jpg]

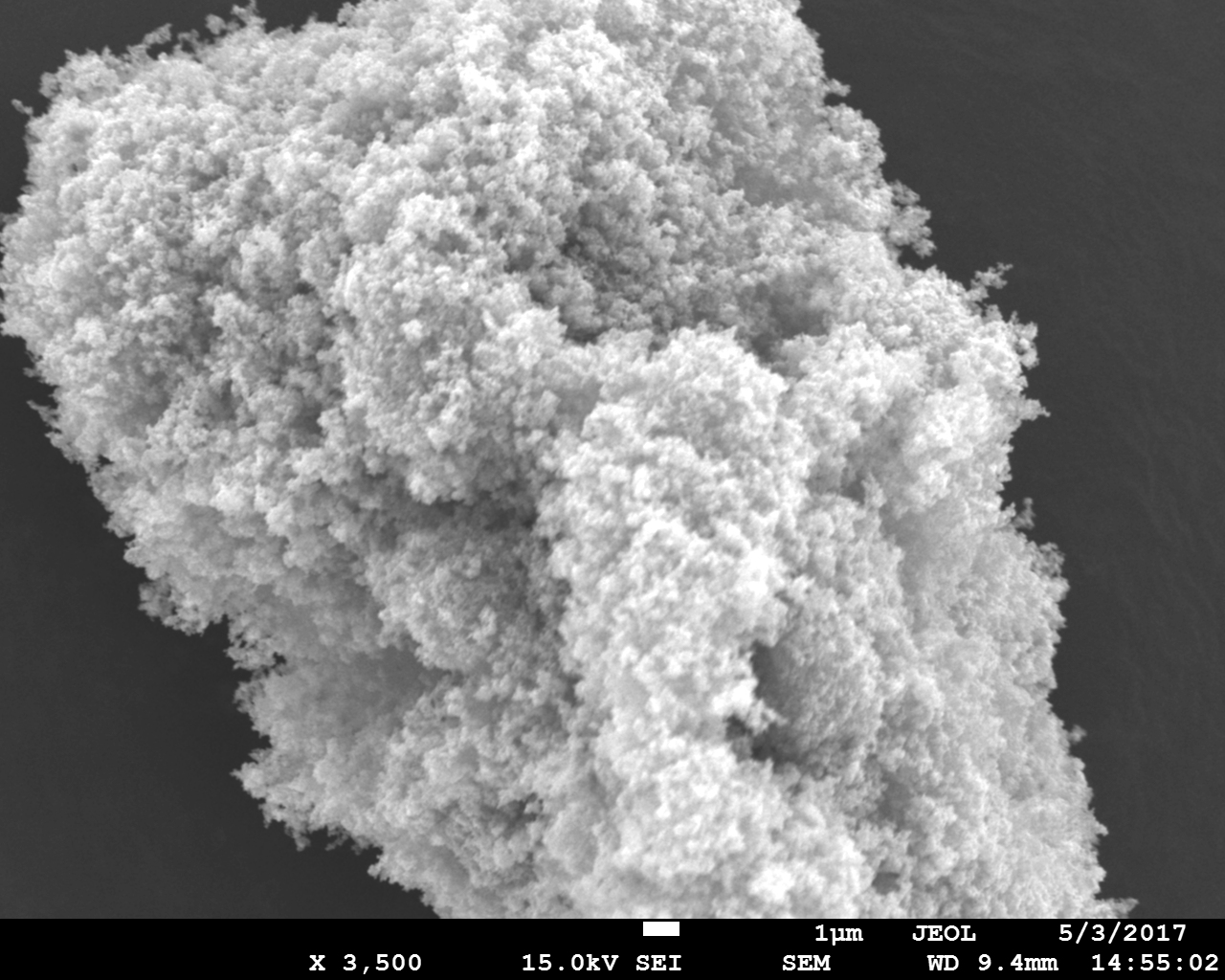

Supplement: Supplemental Information 1 [file peerj-07-6820-s001.zip › Supplemental Files/sem/20170503-18-26/17-1σÉ╕ΘÖäσëì.jpg]

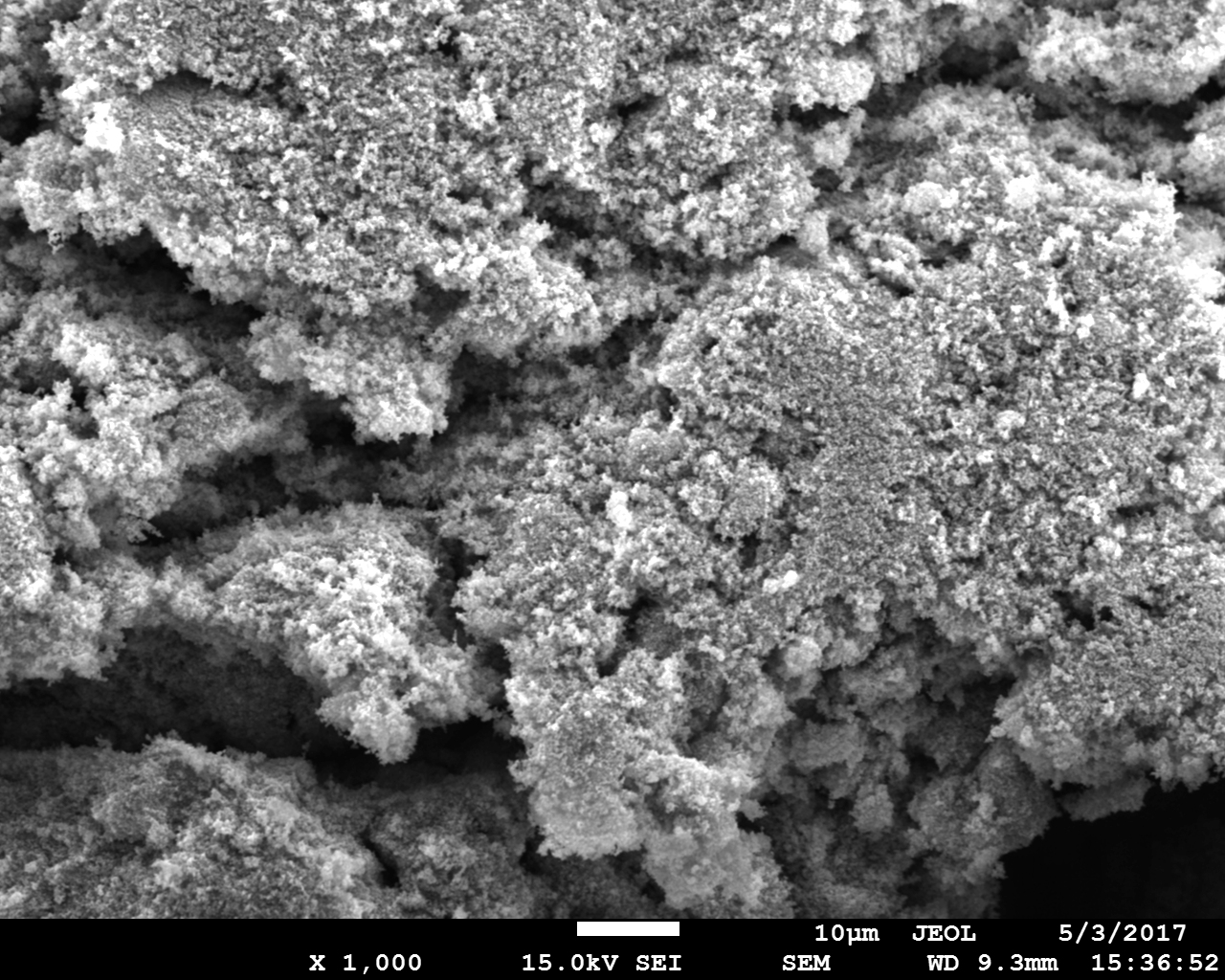

Supplement: Supplemental Information 1 [file peerj-07-6820-s001.zip › Supplemental Files/sem/20170503-18-26/18-3.jpg]

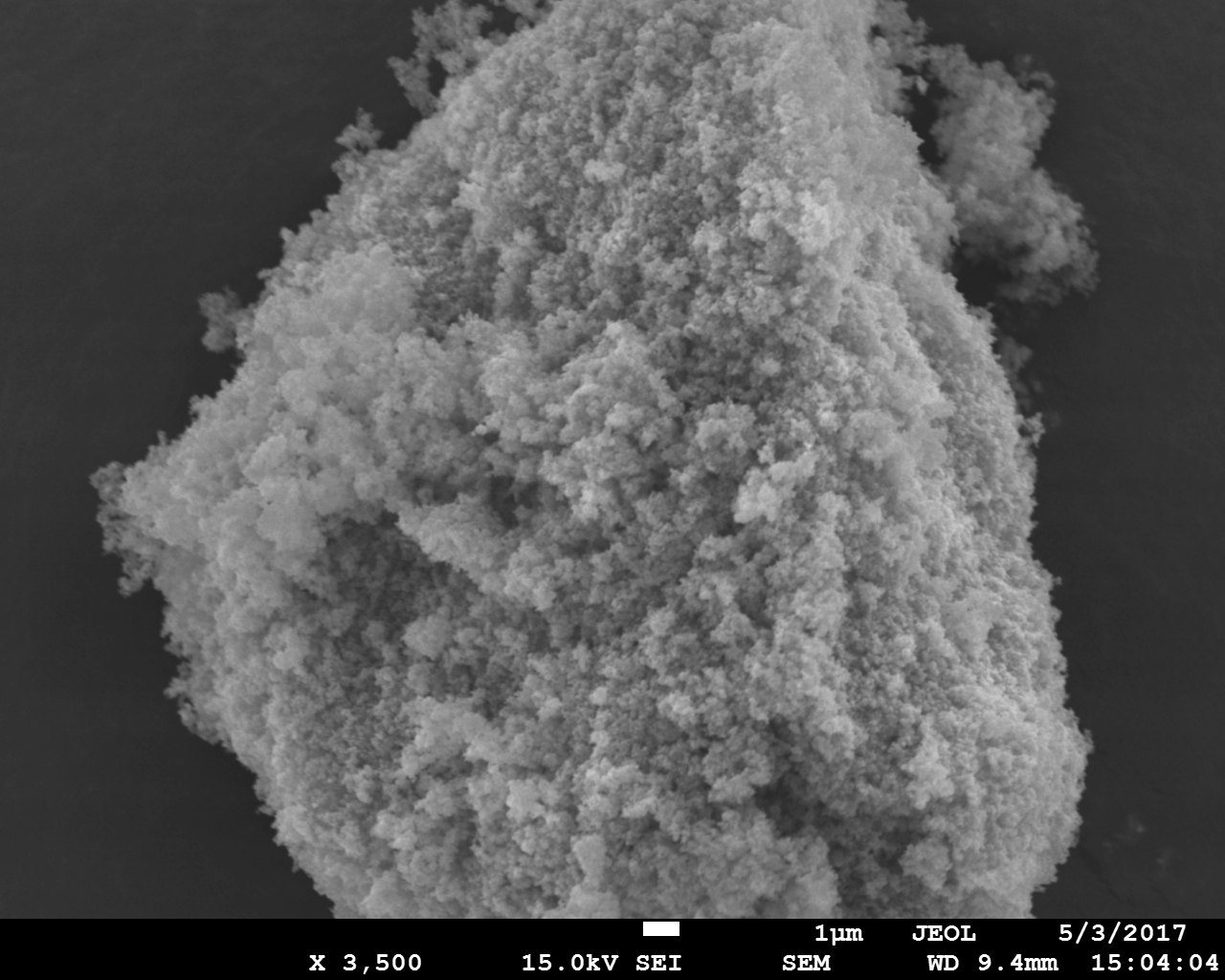

Supplement: Supplemental Information 1 [file peerj-07-6820-s001.zip › Supplemental Files/sem/20170503-18-26/18-1.jpg]

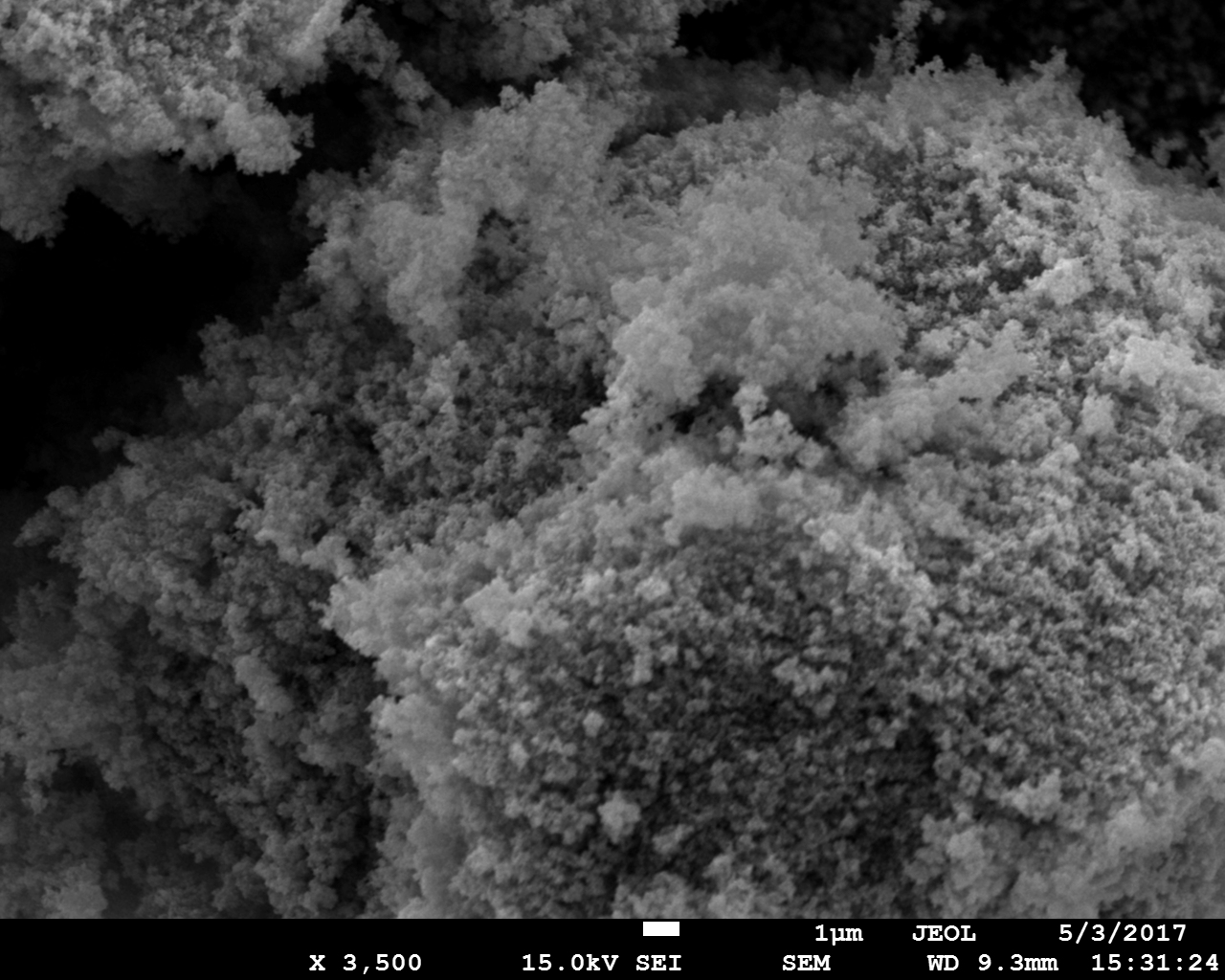

Supplement: Supplemental Information 1 [file peerj-07-6820-s001.zip › Supplemental Files/sem/20170503-18-26/20-2.jpg]

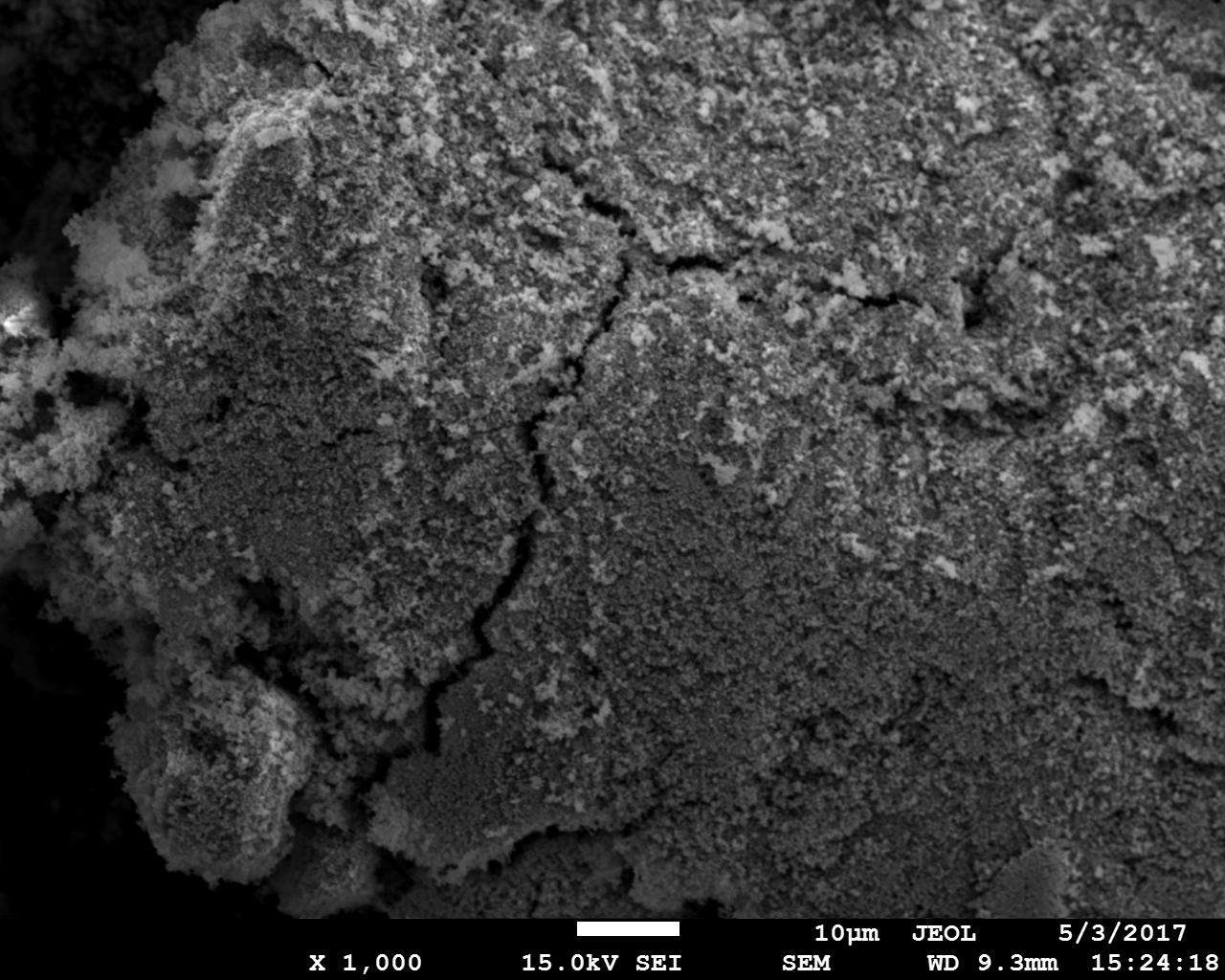

Supplement: Supplemental Information 1 [file peerj-07-6820-s001.zip › Supplemental Files/sem/20170503-18-26/20-1.jpg]

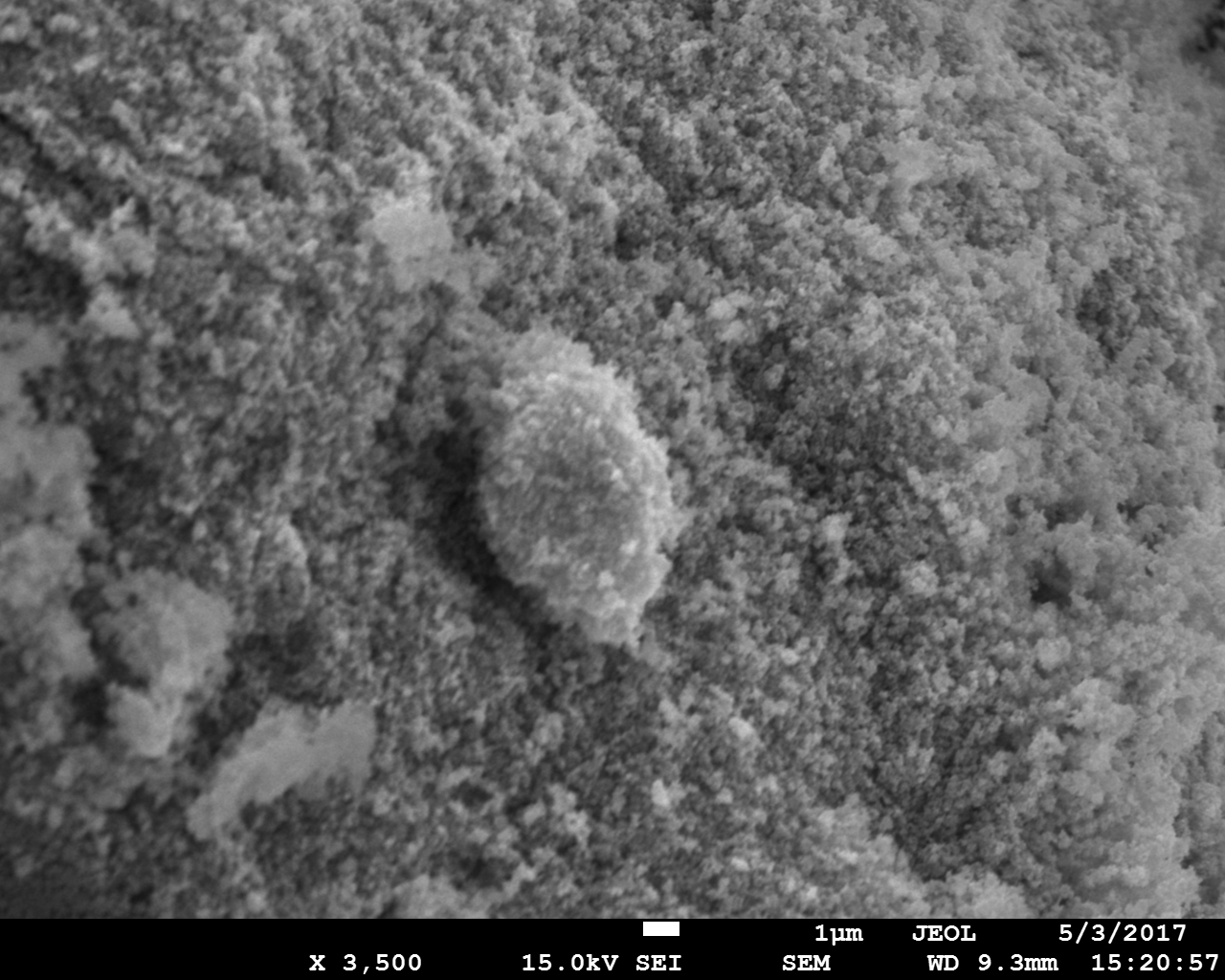

Supplement: Supplemental Information 1 [file peerj-07-6820-s001.zip › Supplemental Files/sem/20170503-18-26/19-1.jpg]

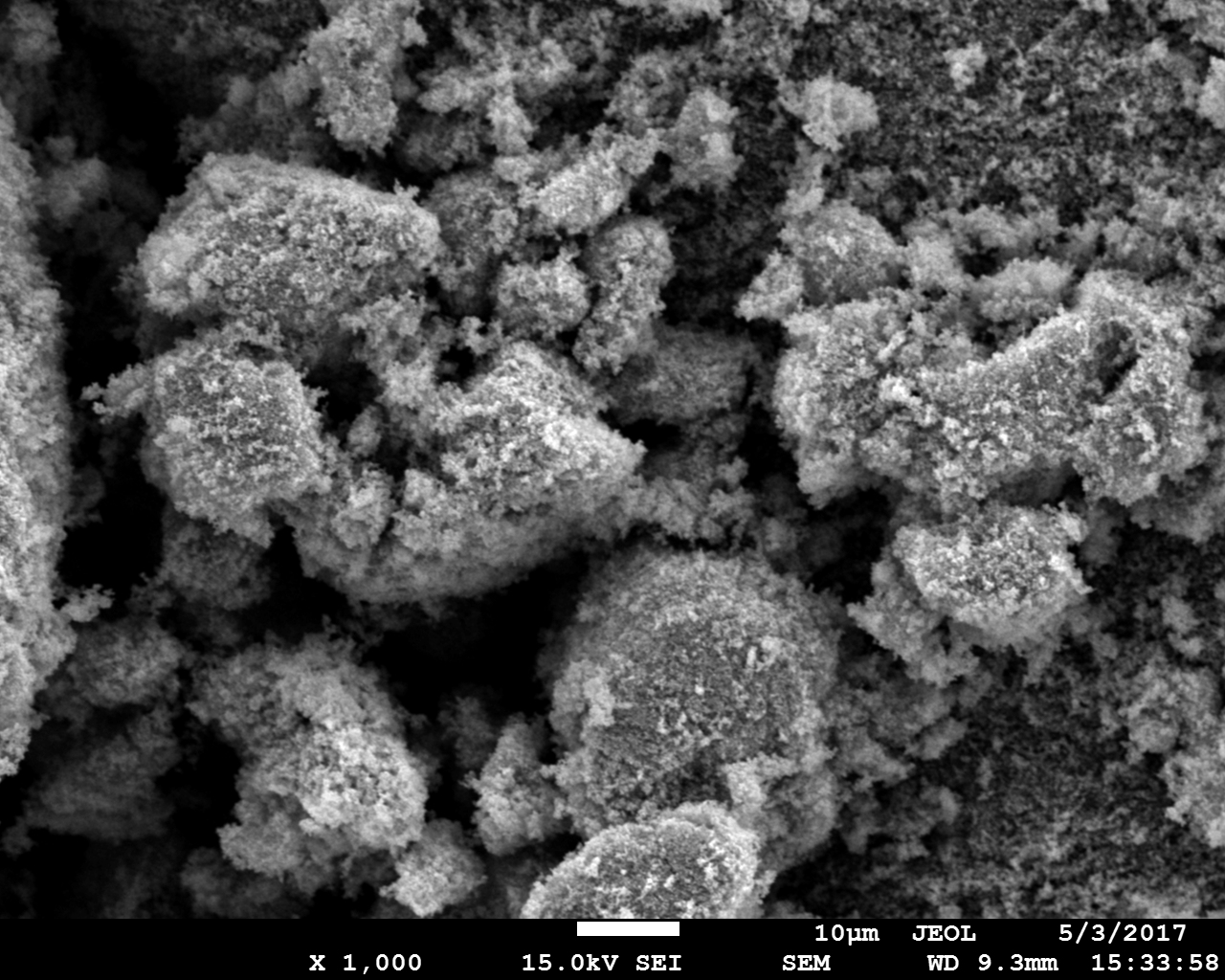

Supplement: Supplemental Information 1 [file peerj-07-6820-s001.zip › Supplemental Files/sem/20170503-18-26/19-2.jpg]
